# Successful first-line treatment of simultaneous multiple primary malignancies of lung adenocarcinoma and renal clear cell carcinoma: A case report

**DOI:** 10.3389/fimmu.2022.956519

**Published:** 2022-08-01

**Authors:** Xiaojun Ye, Xiangliang Liu, Na Yin, Wei Song, Jin Lu, Yi Yang, Xiao Chen

**Affiliations:** Cancer Center, The First Hospital of Jilin University, Changchun, China

**Keywords:** multiple primary malignancies, lung adenocarcinoma, renal clear cell carcinoma, chemotherapy, immunotherapy

## Abstract

**Background:**

Multiple Primary Malignancies (MPMs) refer to the occurrence of two or more primary malignancies in the same organ or multiple organs and tissues of the same patient simultaneously or sequentially, with an incidence rate ranging from 2-17%. According to the difference in the time of occurrence of each primary tumor, MPMs can be classified as simultaneous malignancies and heterochronic malignancies. The former refers to the occurrence of two or more malignancies one after another within 6 months, while the latter refers to the occurrence of two malignancies at an interval of more than 6 months. Currently, there is a lack of effective treatment options for MPMs both nationally and internationally.

**Case presentation:**

The patient was a 65-year-old male smoker with a definite diagnosis of advanced lung adenocarcinoma with kirsten rat sarcoma viral oncogene (KRAS) mutation, concomitant with primary renal clear cell carcinoma (RCCC), who had a progression-free survival (PFS) for 7 months after first-line treatment with albumin-bound paclitaxel and cisplatin in combination with sintilimab.

**Conclusion:**

In this paper, we report a case of advanced lung adenocarcinoma combined with RCCC as a concurrent double primary malignancy, which achieved a satisfactory outcome after first-line chemotherapy combined with immunotherapy, with the aim of exploring effective treatment modalities for this type of MPMs, in order to improve the survival and prognosis of the patient.

## Introduction

Lung cancer is the most common malignant tumor in China, according to the latest data released by China Cancer Center, its morbidity and mortality rank first (1), and non-small cell lung cancer (NSCLC) accounts for about 80-85% of the total number of lung cancers (2). Renal cell carcinoma has a relatively low incidence in China, ranking 14th in incidence and 15th in mortality of malignant tumors (3), among the subtypes, the clear cell type is the most common, accounting for about 70% of renal cell carcinoma (4). While lung cancer combined with primary renal cell carcinoma, only a few cases have been reported (5–7). For such Multiple Primary Malignancies (MPMs), the diagnosis must meet two criteria. First, all tumors must have malignant histologic features. Second, individual tumors must have different pathologic origins, excluding the possibility of metastasis (8). Unfortunately, although diagnostic criteria of MPMs were described at the end of the 19th century (9), there were no guidelines for the treatment of MPMs.

For NSCLC, especially lung adenocarcinoma, the standard first-line chemotherapy is pemetrexed combined with platinum (10). While for patients with low PD-L1 expression, previous studies have shown that the addition of anti-PD-1 inhibitors to standard chemotherapy can significantly improve prognosis (11, 12). Although PD-L1 is an excellent immune marker, it is not a perfect biomarker due to many complex mechanisms. On the one hand, chemotherapy may induce the release of tumor antigens, thereby activating the immune system and enhancing the immune response, playing a synergistic anti-tumor role. On the other hand, due to the temporal and spatial heterogeneity, the level of PD-L1 shown by lung biopsy immunohistochemistry may not be the true level of cancer patients, so NSCLC patients with negative PD-L1 expression may also benefit significantly from immunotherapy. For inoperable renal clear cell carcinoma (RCCC), agents representing the anti-vascular endothelial growth factor or vascular endothelial growth factor receptor pathway, such as sunitinib and sorafenib, have been recommended for first-line therapy (13, 14). But in recent years, combined immunotherapy has shown better efficacy (15). We report a case of first-line application of chemotherapy combined with immunotherapy for advanced lung adenocarcinoma combined with RCCC of a double primary malignancy and propose an individualized treatment approach for this rare case based on the patient’s status, the histological type of the tumor, the stage of the disease and the detection of molecular.

## Case presentation

**Figure 1:**
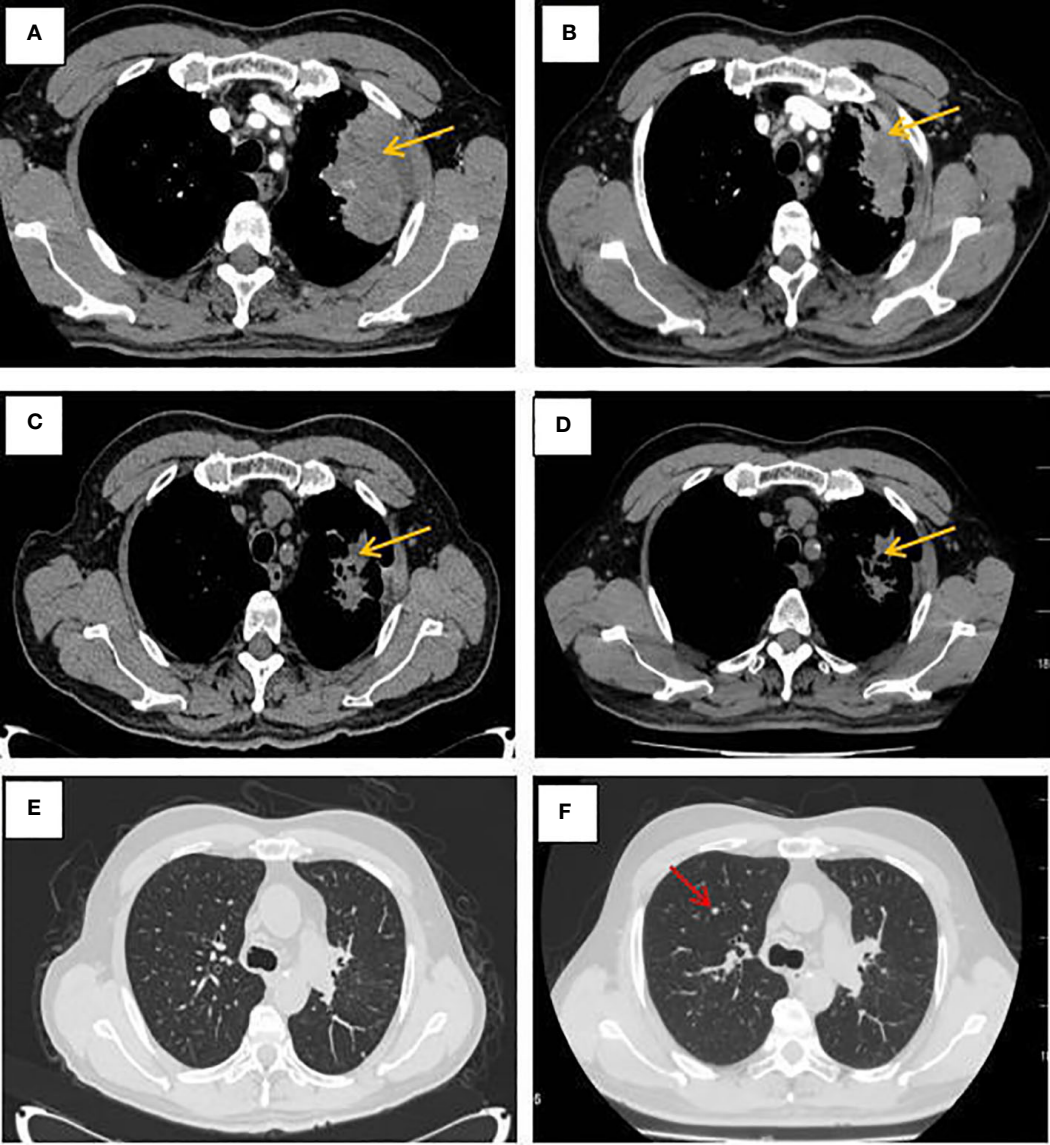
CT of the lungs at different times. **(A–D)** Mediastinal Window; **(E–F)** window of lung fields. **(A)** Pulmonary lesion before treatment. **(B)** CT revealed a stable disease after 2 cycles of albumin-bound paclitaxel and cisplatin in combination with sintilimab. **(C)** CT revealed a stable disease after 6 cycles of albumin-bound paclitaxel and cisplatin in combination with sintilimab. **(D)** CT showed no significant changes in the primary lesion of the left lung after sequential 2 cycles of sintilimab immune monotherapy maintenance treatment. **(E)**The window of lung fields after 6 cycles of albumin-bound paclitaxel and cisplatin in combination with sintilimab. **(F)** CT revealed pulmonary metastases increased after sequential 2 cycles of sintilimab immune monotherapy maintenance treatment (as the red arrow).

**Figure 2:**
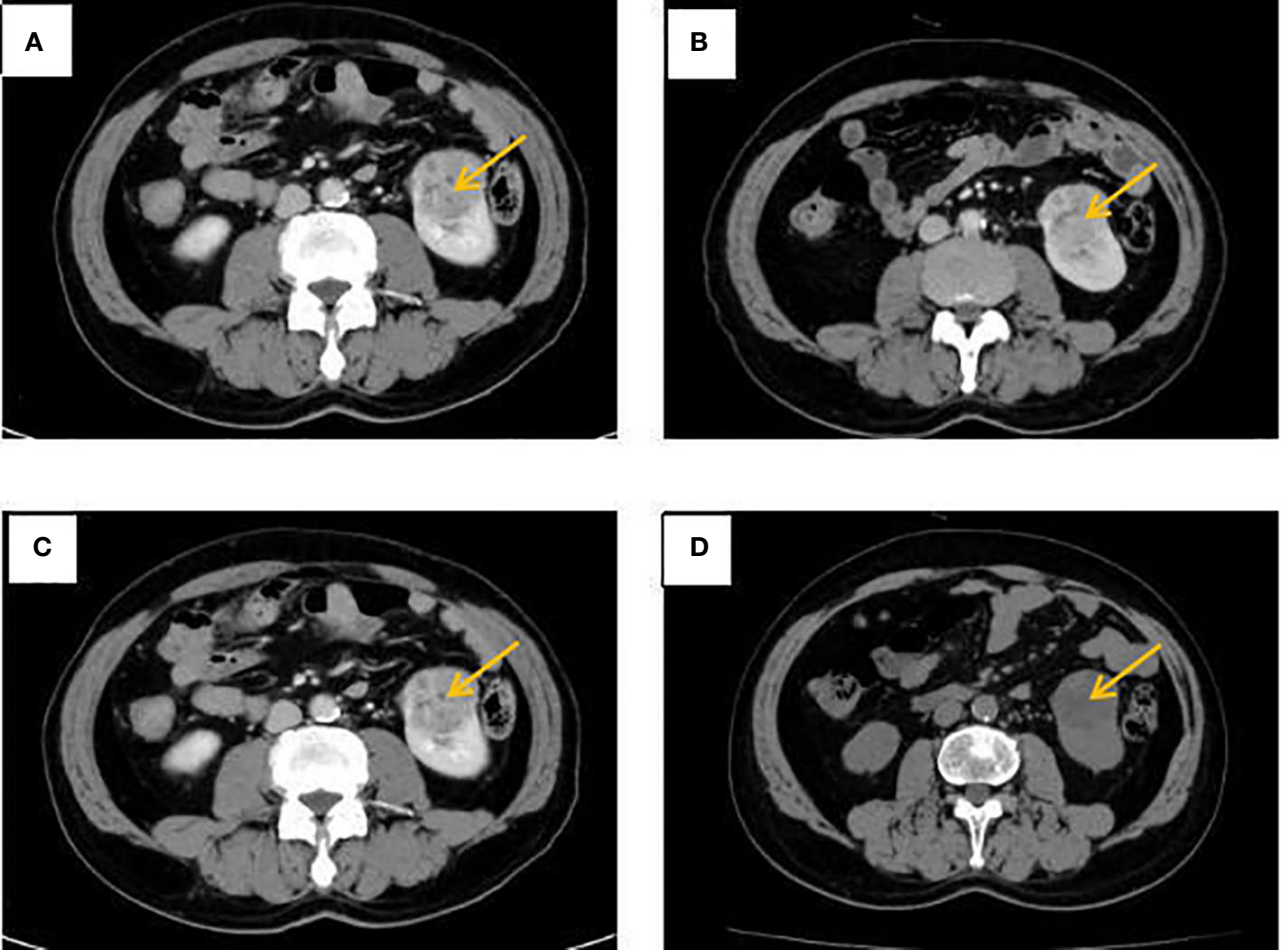
Abdominal CT at different times. **(A)** The left kidney lesion before treatment. **(B)** CT revealed a stable disease after 2 cycles of albumin-bound paclitaxel and cisplatin in combination with sintilimab. **(C)** CT revealed a stable disease after 6 cycles of albumin-bound paclitaxel and cisplatin in combination with sintilimab. **(D)** No significant change in left kidney lesion after sequential 2 cycles of sintilimab immune monotherapy maintenance treatment.

**Figure 3:**
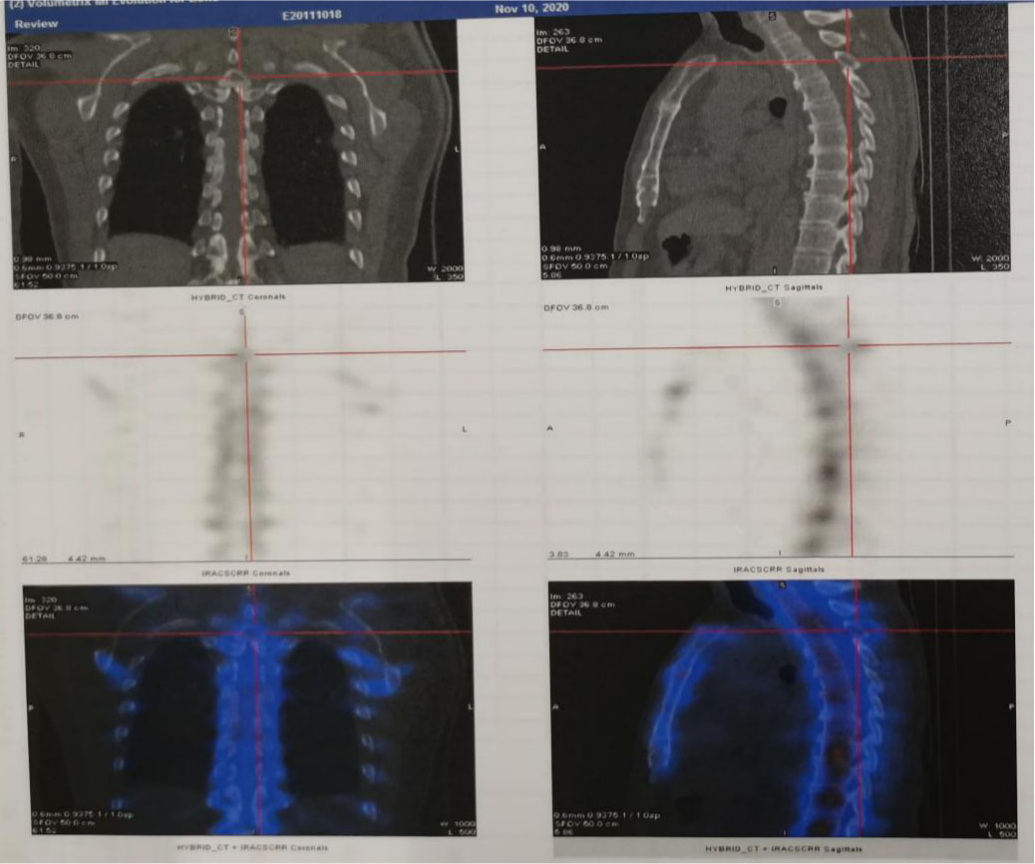
Whole body bone imaging showed increased radioactivity in the third thoracic vertebra with bone destruction.

**Figure 4:**
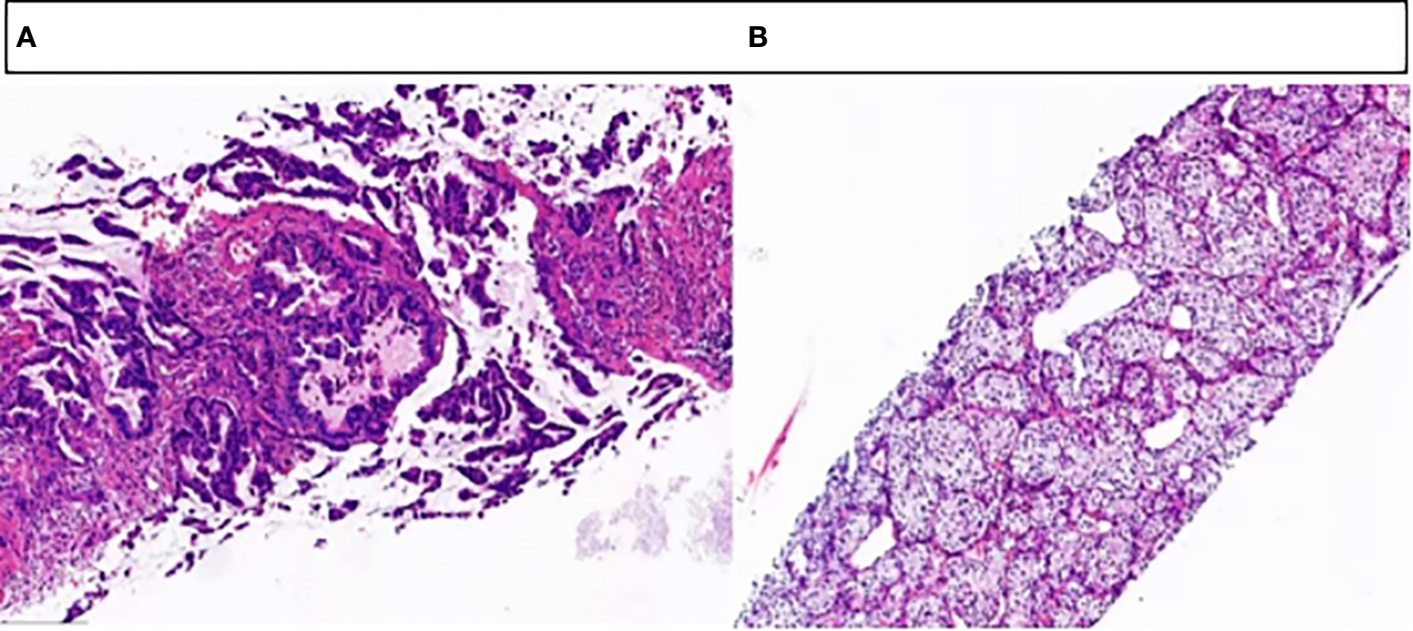
Tissue sections stained with hematoxylin-eosin. **(A)** Adenocarcinoma of the left lung. **(B)** Left renal clear cell carcinoma.

**Figure 5:**
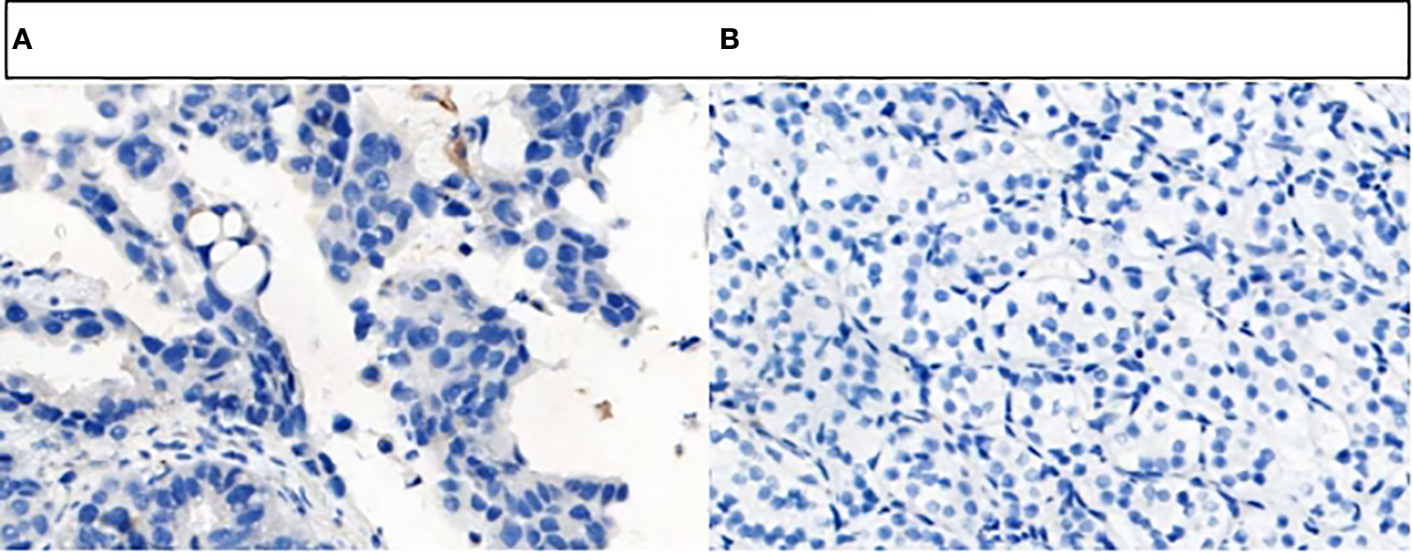
PD-L1 protein expression on tumor cells was detected by VENTATA PD-L1(SP263). **(A)** The results was negative. **(B)** The results was negative.

The patient, a 65-year-old male, was admitted to the hospital for the first time on November 6, 2020, with “cough, chest tightness and chest and back pain for 1 month”. He had a history of smoking for 40 years, with about 5 cigarettes per day. Computed tomography (CT) of the lungs suggested occupancy of the upper lobe of the left lung, about 9.4x5.4cm in size, and invasion of the adjacent left rib pleura, mediastinal and left hilar lymph node enlargement, multiple nodules of 0.3-0.8cm in both lungs (**Figure 1A**). Abdominal CT suggested occupancy in the lower pole of the left kidney with 5.0cm in size (**Figure 2A**). Bone scan suggested metastasis to the 3rd thoracic vertebra (**Figure 3**), while ultrasound and head CT of the remaining cervical lymph nodes showed no abnormality. The lung puncture biopsy revealed adenocarcinoma, and immunohistological staining results showed positive for cytokeratin 7 (CK7^+^), cell proliferation antigen Ki-67(50%^+^), thyroid transcription factor 1 (TTF-1^+^), Napsin A^+^, negative for cytokeratin 20 (CK20^-^), cytokeratin 5/6 (CK5/6^-^), tumor suppressor gene P40^-^ (**Figure 4A**). Renal puncture biopsy suggested RCCC, and immunohistochemistry showed pan cytokeratin (CK-pan^+^), cytokeratin 7 (CK7^+^), anticytokeratin (CAM5.2^+^), Vimentin^+^, alpha-methylacyl CoA racemase (P504S^+^), paired box protein-8 (PAX-8^+^), cluster of differentiation 10 (CD10^+^), cluster of differentiation 117 (CD117^-^), cytokeratin 20 (CK20^-^), transcription factor E3 (TFE3^-^) (**Figure 4B**). Genetic testing of lung tissues showed point mutations in exon 2 of KRAS gene, and programmed death-ligand 1 (PD-L1) protein expression tests in both lung and kidney tissues were negative (**Figures 5A, B**).

**Figure 6:**
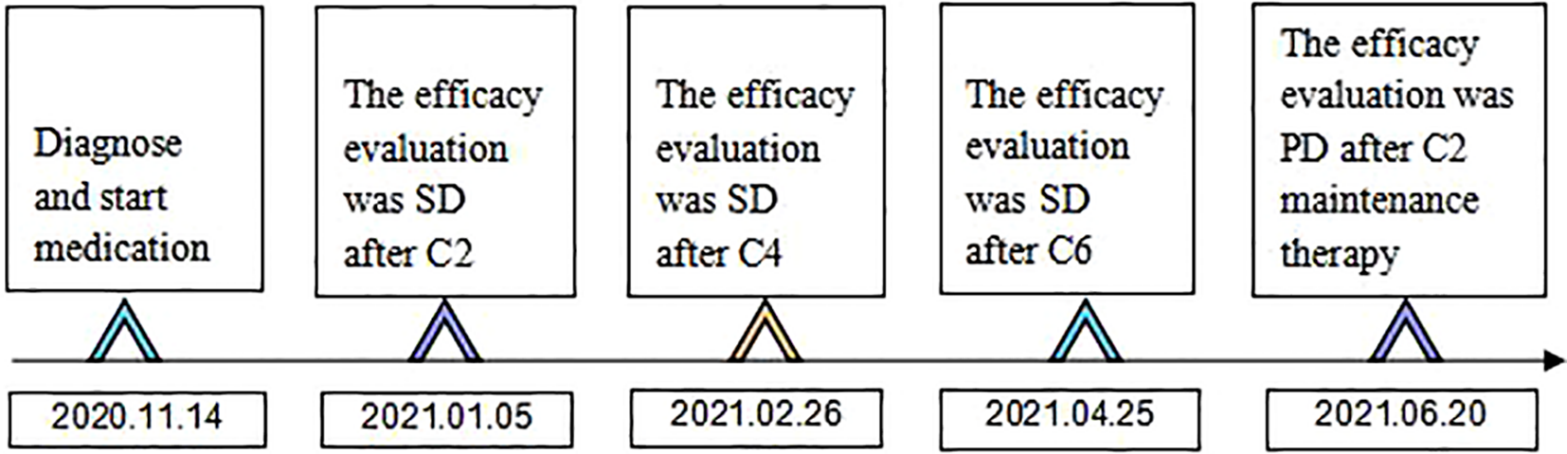
Time flow chart of the diagnosis and treatment process of this patient. (C2 represents 2 courses of treatment, C4 represents 4 courses of treatment, C6 represents 6 courses of treatment, C2 maintenance therapy represents 2 courses of maintenance therapy).

Combined with symptoms, imaging examination and pathological findings, the patient was clinically diagnosed as left lung adenocarcinoma (cT4N2M1c, stage IVb, KRAS^+^, EGFR^-^, ALK^-^, ROS1^-^, PD-L1^-^), multiple metastases in both lungs, bone metastases, and clear cell carcinoma of the left kidney (cT1bN0M0 stage I, PD-L1^-^). Among them, lung cancer and RCCC staging were defined according to the TNM staging system, Edition 8 of the American Joint Committee on Cancer (AJCC). He was treated with albumin-bound paclitaxel and cisplatin in combination with sintilimab in one cycle every 21 days (the treatment flow chart was shown in **Figure 6**). After 2 cycles, the patient self-reported symptoms of cough and chest tightness were significantly relieved. According to the Response Evaluation Criteria in Solid Tumors 1.1 (RECIST 1.1), chest CT scan confirmed a stable disease (SD, **Figure 1B**), and the left kidney lesion was assessed as SD (**Figure 2B**). Continuing the treatments of albumin-bound paclitaxel and cisplatin combined with sintilimab, the efficacy of pulmonary lesions and renal lesions were still evaluated as SD after 6 cycles of treatment (**Figures 1C**, **2C**). Subsequently, the follow-up examination on June 20, 2021, found that after 2 cycles of immune monotherapy maintenance therapy, there were no significant changes in the primary lesion of the left lung (**Figure 1D**), but new lung metastases occurred (**Figures 1F** vs. **1E**). While the left renal lesion was similar to the previous lesion (**Figure 2D**). Overall, the disease was evaluated as progress.

## Discussion

In this case, the immunohistochemical results of CK7, Napsin A and TTF-1 supporting the diagnosis of lung adenocarcinoma were all positive, and the immunohistochemical results of Vimentin, CD10 and PAX-8 supporting the diagnosis of RCCC were all positive. Combined with the morphology and immunohistochemical results of tumor tissue, it was clear that the two tumors of this patient occurred independently, not by metastasis. Currently, the pathogenesis of MPMs is still unclear and generally considered to be the result of the long-term effect of multiple carcinogenic factors, which are related to genetic factors, intrinsic factors (susceptibility, immune status, endocrine), physical and chemical environment (long-term exposure to radiation and industrial pollution) and bad lifestyle (smoking, alcohol abuse), etc. (6, 16–18). The patient in this case was a long-term smoker, which is not only a high risk factor for the development of lung cancer, but also a common causative factor for RCCC. A potential association between smoking and KRAS mutations has been shown, with a 25-35% incidence of KRAS mutations in smokers compared to 5% in nonsmokers (19). Gene detection of this patient’s lung tissue revealed KRAS mutations, suggesting that smoking may promote the expression of genetic susceptibility that together leads to the development of MPMs. In addition, some research point out (20), KRAS mutation is a more common molecular alteration in advanced NSCLC and associated with poor prognosis. Therefore, it is crucial to formulate an individualized treatment plan for the synchronous double primary malignancy with KRAS mutation in this case, with late staging and poor prognosis.

It has been stated that the choice of treatment for multiple cancers should depend on the potential malignancy of each primary tumor, with priority given to tumors with higher primary malignancy and more advanced staging to develop treatment plans (7). Compared with RCCC, lung adenocarcinoma is prone to distant metastasis to the skull, liver, bone, and adrenal glands (21), whereas RCCC has a lower malignancy and relatively few distant metastasis (22). In this case, the lung adenocarcinoma had a larger tumor load, higher malignancy, and more advanced stage, therefore, bone metastasis was considered to originate from pulmonary lesions. In view of the above considerations, priority needs to be given to lung cancer in the treatment, while taking into account the treatment of kidney tumors. Based on the results of the Keynote-189 study (23), the addition of pembrolizumab to standard chemotherapy with pemetrexed and platinum significantly prolonged progression-free survival (PFS) and overall survival (OS) in previously untreated patients with advanced non-squamous NSCLC without EGFR or ALK mutations, regardless of PD-L1 expression levels. Subsequently, the ORIENT-11 study (24) continued the treatment paradigm of immunotherapy combined with chemotherapy by applying a selective anti-PD-1 monoclonal antibody, sintilimab, to exert anti-tumor effects by blocking the PD-1/PD-L1 interaction to reactivate immune cells, and showed that the addition of sintilimab to standard chemotherapy significantly prolonged median PFS by 4.2 months (9.2 vs. 5.0 months), which was similar to the results of the Keynote-189 study (median PFS prolongation of 4.1 months, 9.0 vs. 4.9 months). These studies confirmed the role of immunotherapy in the first-line treatment of locally advanced or metastatic non-squamous NSCLC.

In addition, one study reported that the presence of driver gene mutations may be an independent factor affecting the prognosis of NSCLC (25). In particular, patients with G12C mutation have a mutation rate of 13% in lung adenocarcinoma (26). Sotorasib was the first inhibitor of KRAS G12C mutation, and the CodeBreak 100 phase II study showed that mPFS was 6.8 months (26). On May 58, 2021, the US Food and Drug Administration has granted accelerated approval to sotorasib for the treatment of adults with advanced NSCLC with KRAS G12C mutation (27). But considering that the patient in this case was diagnosed on November 14, 2020, there are currently no approved KRAS mutation-targeting drugs available at this time. Therefore, primers for KRAS specific mutation sites were not designed for the ten genes detected by Polymerase Chain Reaction (PCR) technology. However, it is worth noting that patients with KRAS mutations showed a better response to PD-1 inhibitors, and PFS and OS are also improved (28, 29). It has been claimed that KRAS mutations promote T cell infiltration and enhance tumor immunogenicity, thereby improving the immune efficacy of PD-1/PD-L1 inhibitors (30). Genetic testing of lung tissue in this patient showed a point mutation in exon 2 of KRAS, and the use of PD-1 inhibitors may have a favorable prognosis for the patient. Ultimately, the patient chose immunotherapy with sintilimab.

On the other hand, given the negative PD-L1 expression in lung and kidney tissues of this case, chemotherapy combined with immunotherapy may achieve better efficacy. Chemotherapy is a routine treatment for malignant tumors, but although chemotherapy has certain effects, it largely lacks the tumor killing effect of bystander, and at tolerable doses, the remaining malignant cells can easily escape and become resistant (31). However, chemotherapy-induced death of cancer cells can release a large number of cytoplasmic, nuclear proteins and other substances, which can improve the immunogenicity of cancer cells, transform immune “cold” tumors into immune “hot” tumors, and ultimately promote the recognition and elimination of residual cancer cells by immune checkpoint inhibitors (32–34). Therefore, chemotherapy combined with immunotherapy has a certain synergistic antitumor effect.

The standard first-line chemotherapy regimen for patients with advanced NSCLC is a platinum-based agent. Pemetrexed is recommended for patients with NSCLC, particularly non-squamous cell carcinoma (35, 36), but paclitaxel has broader anticancer activity that inhibits tumor cell mitosis by promoting microtubulin polymerization and inhibiting its depolymerization, this mechanism that makes paclitaxel potentially efficacious in renal cell carcinoma as well (37, 38). Albumin-bound paclitaxel is a novel solvent-free formulation of paclitaxel that reduces the risk of hypersensitivity reactions and hematotoxicity caused by organic solvents (39). Therefore, albumin-bound paclitaxel in combination with cisplatin was chosen for chemotherapy. Of note, albumin-bound paclitaxel and cisplatin also have toxic and side effects, including bone marrow suppression, extremities numbness caused by peripheral neuropathy, renal toxicity and gastrointestinal reactions (40, 41). In this case, chemotherapy-related bone marrow suppression occurred during the treatment. After the third course of treatment, the platelet decreased to 80x10^9^/L (normal range is 100-300x10^9^/L), but the platelet returned to normal after the treatment with thrombopoietin. In addition to chemotherapy and immunotherapy, anti-angiogenic drugs such as bevacizumab have also been proven to have good therapeutic effects (42). However, considering the economic status of the patient and the strong toxicity of the combination of four drugs, the family finally chose not to combine the anti-angiogenesis drugs.

**Figure 7:**
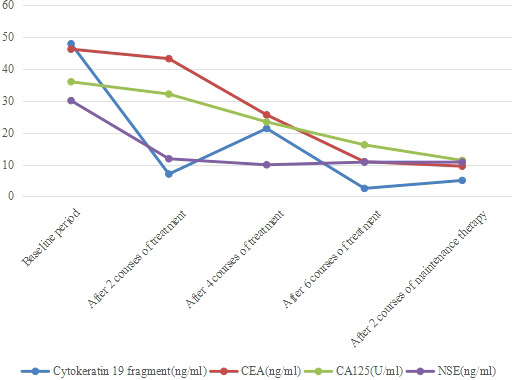
Changes in tumor marker content. Cytokeratin 19 fragment, CEA, CA125 and NSE were abnormally increased before treatment, after 2 courses of treatment, cytokeratin 19 fragment and NSE decreased significantly. After 6 courses of treatment, CEA and CA125 also decreased significantly.

Surprisingly, the patient’s tumor markers continued to decrease during treatment. In the baseline period, cytokeratin 19 fragment, carcinoembryonic antigen (CEA), carbohydrate antigen 125(CA125) and neuron specific enolase (NSE) were abnormally increased, especially cytokeratin 19 fragment and CEA (**Figure 7**). After 2 courses of treatment, CA125 and NSE were reduced to normal, and after 6 courses of treatment, cytokeratin 19 fragment was reduced to normal, and CEA was also significantly reduced, indicating that albumin-bound paclitaxel and cisplatin in combination with sintilimab can effectively control the growth of tumor cells.

**Figure 8:**
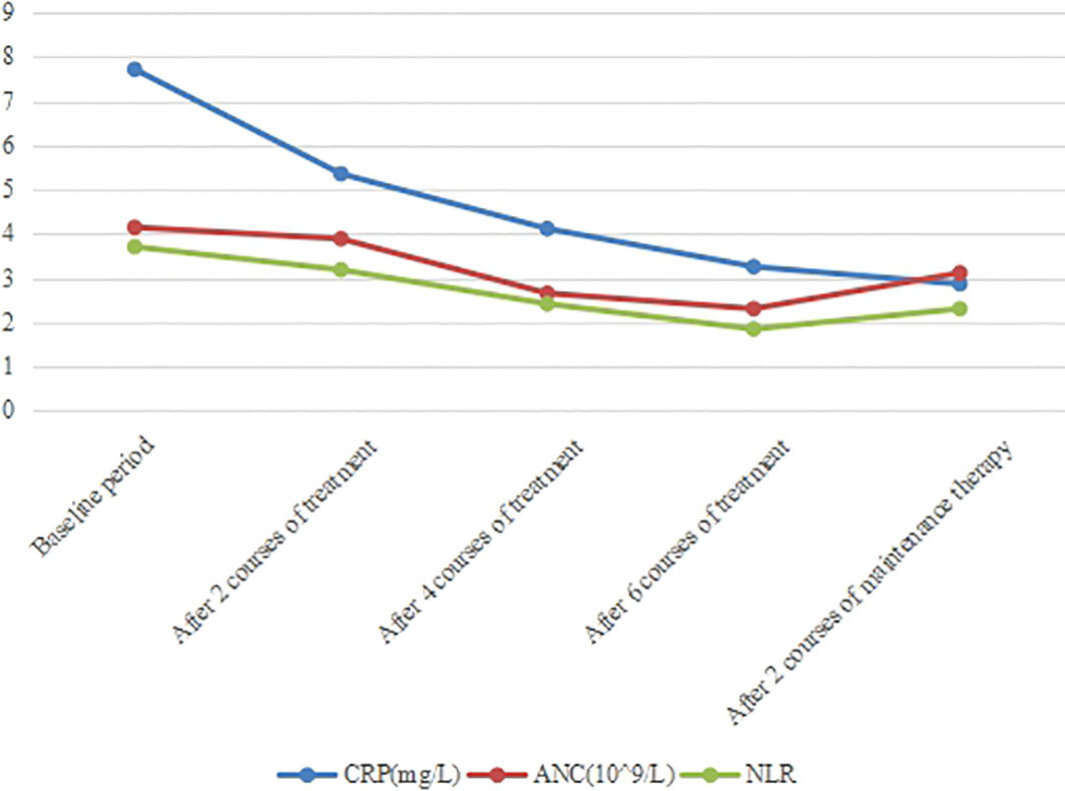
Changes of peripheral blood inflammatory indexes. The content of ANC, CRP and the ratio of NRL gradually decreased after treatment.

**Figure 9:**
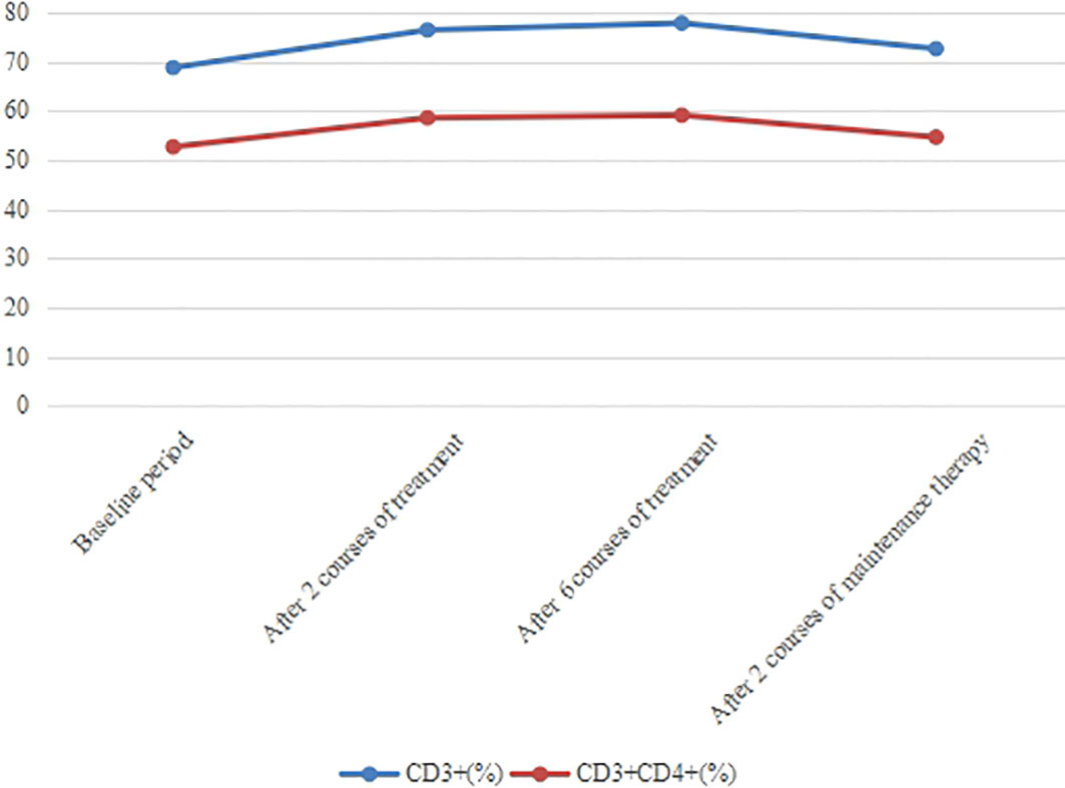
Changes in the proportion of T cells in peripheral blood. With the treatment progressed, the content of CD3^+^ and CD3^+^CD4^+^ increased until the end of 6 courses of treatment.

In recent years, great progress has been made in the exploration of the relationship between inflammation and cancer. Inflammatory response plays an important role in the occurrence and development of tumors (43). For example, neutrophil count (ANC), C-reactive protein (CRP), neutrophil-to-lymphocyte ratio (NLR) can reflect the inflammatory state of the body (44–46). Studies have shown that there is a negative correlation between neutrophils and CD8^+^ T cell content in NSCLC (47). Theoretically, T lymphocytes reflect cell-mediated immune responses and play an important role in anti-tumor immune responses (48, 49). Among them, the change of CD3^+^ T lymphocyte number represents the change of total T lymphocyte ratio in peripheral blood. Neutrophils represent a response to systemic inflammation, suggesting that neutrophils are involved in an inflammatory response that inhibits anti-tumor immune responses by inhibiting the cytotoxic activity of immune cells, especially activated T cells (50). Therefore, reduced NLR and elevated T lymphocytes may be associated with better response to immunotherapy and prognosis in patients with advanced cancer. In this case, ANC, CRP and NLR were all within the normal range before treatment, and further reduced after 2 and 6 courses of treatment (**Figure 8**). Moreover, the content of CD3^+^ and helper/inducer T cells (CD3^+^CD4^+^) increased after treatment (**Figure 9**), revealing that these peripheral blood indicators may be closely related to the good prognosis of this patient.

## Conclusion

In this case, the PFS of the patient was 7 months for first-line treatment with chemotherapy (albumin-bound paclitaxel and cisplatin) combined with immunotherapy (sintilimab), which significantly improved the survival of the patients. In addition, no significant changes were observed in the renal lesions, indicating that chemotherapy combined with immunotherapy could better control renal tumor growth. Therefore, for MPMs, first, it is necessary to identify the lesion in the formulation of the treatment, and give priority to the tumor with a higher degree of malignancy, while taking into account the other tumor. Second, for NSCLC with KRAS mutation and RCCC with relatively low malignancy, despite negative PD-L1 expression, albumin-bound paclitaxel and platinum chemotherapy combined with PD-1 monoclonal antibody immunotherapy is an effective treatment option.

## Data availability statement

The original contributions presented in the study are included in the article/supplementary material. Further inquiries can be directed to the corresponding author.

## Ethics statement

Written informed consent was obtained from the individual(s) for the publication of any potentially identifiable images or data included in this article.

## Author contributions

XY obtained the computed tomography images and drafted the manuscript. NY and YY reviewed literature data. XY, JL, WS, and XC participated in the conception, the design of the study. XL and XC carried out critical revision. All authors have read and agreed to the published version of the manuscript.

## Conflict of interest

The authors declare that the research was conducted in the absence of any commercial or financial relationships that could be construed as a potential conflict of interest.

## Publisher’s note

All claims expressed in this article are solely those of the authors and do not necessarily represent those of their affiliated organizations, or those of the publisher, the editors and the reviewers. Any product that may be evaluated in this article, or claim that may be made by its manufacturer, is not guaranteed or endorsed by the publisher.

## References

[1] Zheng R Zhang S Zeng H Wang S Sun K He J. Cancer incidence and mortality in China, 2016. J Natl Cancer Center (2022). doi: 10.1016/j.jncc.2022.02.002 PMC11256658 39035212

[2] Friedlaender A Addeo A Russo A Gregorc V Cortinovis D Rolfo CD. Targeted therapies in early stage NSCLC: hype or hope? Int J Mol Sci (2020) 21(17):6329. doi: 10.3390/ijms21176329 PMC7504271 32878298

[3] Marostica E Barber R Denize T Kohane IS Signoretti S Golden JA. Development of a histopathology informatics pipeline for classification and prediction of clinical outcomes in subtypes of renal cell carcinoma. Clin Cancer Res (2021) 27(10):2868–78. doi: 10.1158/1078-0432.CCR-20-4119 33722896

[4] Jonasch E Walker CL Rathmell WK. Clear cell renal cell carcinoma ontogeny and mechanisms of lethality. Nat Rev Nephrol (2021) 17(4):245–61. doi: 10.1038/s41581-020-00359-2 PMC8172121 33144689

[5] Sato S Shinohara N Suzuki S Harabayashi T Koyanagi T. Multiple primary malignancies in Japanese patients with renal cell carcinoma. Int J Urol (2004) 11(5):269–75. doi: 10.1111/j.1442-2042.2004.00792.x. 15147541

[6] Sakellakis M Peroukides S Iconomou G Boumpoucheropoulos S Kalofonos H. Multiple primary malignancies: a report of two cases. Chin J Cancer Res (2014) 26(2):215–8. doi: 10.3978/j.issn.1000-9604.2014.02.15 PMC4000907 24826064

[7] Mazouz A Amaadour L Souaf I El Fatemi H Amarti A Erraisse MA. Synchronous malignant renal mass in patient with a lung cancer: case report and literature review. Pan Afr Med J (2015) 20:22. doi: 10.11604/pamj.2015.20.22.5541 26015842 PMC4432812

[8] Warren S Gates O. Multiple malignant tumors. a survey of the literature and statistical study. Am J Cancer (1932) 16:1358–414. doi: 10.1016/0016-5085(87)90440-9

[9] Billroth T. Die allgemeine chirurgische pathologie und therapie. 51 vorlesungen. In: Ein handbuch fur studierende and artze. Berlin: G Reimer (1889). p. 934–53.

[10] Paz-Ares LG de Marinis F Dediu M Thomas M Pujol JL Bidoli P. PARAMOUNT: Final overall survival results of the phase III study of maintenance pemetrexed versus placebo immediately after induction treatment with pemetrexed plus cisplatin for advanced nonsquamous non-small-cell lung cancer. J Clin Oncol (2013) 31(23):2895–902. doi: 10.1200/JCO.2012.47.1102 23835707

[11] Mok TSK Wu YL Kudaba I Kowalski DM Cho BC Turna HZ. Pembrolizumab versus chemotherapy for previously untreated, PD-L1-expressing, locally advanced or metastatic non-small-cell lung cancer (KEYNOTE-042): a randomised, open-label, controlled, phase 3 trial. Lancet (2019) 393(10183):1819–30. doi: 10.1016/S0140-6736(18)32409-7 30955977

[12] Liu L Bai H Wang C Seery S Wang Z Duan J. Efficacy and safety of first-line immunotherapy combinations for advanced NSCLC: a systematic review and network meta-analysis. J Thorac Oncol (2021) 16(7):1099–117. doi: 10.1016/j.jtho.2021.03.016 33839365

[13] Motzer RJ Hutson TE Tomczak P Michaelson MD Bukowski RM Oudard S. Overall survival and updated results for sunitinib compared with interferon alfa in patients with metastatic renal cell carcinoma. J Clin Oncol (2009) 27(22):3584–90. doi: 10.1200/JCO.2008.20.1293 PMC3646307 19487381

[14] McCormack PL. Pazopanib: a review of its use in the management of advanced renal cell carcinoma. Drugs (2014) 74(10):1111–25. doi: 10.1007/s40265-014-0243-3 24935162

[15] Bedke J Albiges L Capitanio U Giles RH Hora M Lam TB. The 2021 updated European association of urology guidelines on renal cell carcinoma: immune checkpoint inhibitor-based combination therapies for treatment-naive metastatic clear-cell renal cell carcinoma are standard of care. Eur Urolc (2021) 80(4):393–7. doi: 10.1016/j.eururo.2021.04.042 34074559

[16] Beisland C Talleraas O Bakke A Norstein J. Multiple primary malignancies in patients with renal cell carcinoma: a national population-based cohort study. Bju Int (2006) 97(4):698–702. doi: 10.1111/j.1464-410X.2006.06004.x 16536756

[17] Takalkar U Asegaonkar BN Kodlikeri P Asegaonkar S Sharma B Advani SH. An elderly woman with triple primary metachronous malignancy: A case report and review of literature. Int J Surg Case Rep (2013) 4(7):593–6. doi: 10.1016/j.ijscr.2013.03.032 PMC3679429 23702365

[18] Wood ME Vogel V Ng A Foxhall L Goodwin P Travis LB. Second malignant neoplasms: assessment and strategies for risk reduction. J Clin Oncol (2012) 30(30):3734–45. doi: 10.1200/JCO.2012.41.8681 23008293

[19] Mao C Qiu LX Liao RY Du FB Ding H Yang WC. KRAS mutations and resistance to EGFR-TKIs treatment in patients with non-small cell lung cancer: a meta-analysis of 22 studies. Lung Cancer (2010) 69(3):272–8. doi: 10.1016/j.lungcan.2009.11.020 20022659

[20] Song P Yang D Wang H Cui X Si X Zhang X. Relationship between the efficacy of immunotherapy and characteristics of specific tumor mutation genes in non-small cell lung cancer patients. Thorac Cancer (2020) 11(6):1647–54. doi: 10.1111/1759-7714.13447 PMC7262886 32342665

[21] Munoz MG Ovejero DA Gorospe SL. Surgical approach of non-small cell lung cancer with extrapulmonary metastasis. Med Clin (Barc) (2019) 153(3):115–21. doi: 10.1016/j.medcli.2019.02.025 31151683

[22] Chen J Cao N Li S Wang Y. Identification of a risk stratification model to predict overall survival and surgical benefit in clear cell renal cell carcinoma with distant metastasis. Front Oncol (2021) 11:630842. doi: 10.3389/fonc.2021.630842 33777784 PMC7991397

[23] Gadgeel S Rodríguez-Abreu D Speranza G Esteban E Felip E Dómine M. Updated analysis from KEYNOTE-189: pembrolizumab or placebo plus pemetrexed and platinum for previously untreated metastatic nonsquamous non-small-cell lung cancer. J Clin Oncol (2020) 38(14):1505–17. doi: 10.1200/JCO.19.03136 32150489

[24] Yang Y Sun J Wang Z Fang J Yu Q Han B. Updated overall survival data and predictive biomarkers of sintilimab plus pemetrexed and platinum as first-line treatment for locally advanced or metastatic nonsquamous NSCLC in the phase 3 ORIENT-11 study. J Thorac Oncol (2021) 16(12):2109–20. doi: 10.1016/j.jtho.2021.07.015 34358724

[25] Ono A Isaka M Serizawa M Omae K Kojima H Nakashima K. Genetic alterations of driver genes as independent prognostic factors for disease-free survival in patients with resected non-small cell lung cancer. Lung Cancer (2019) 128:152–7. doi: 10.1016/j.lungcan.2018.12.005 30553548

[26] Zhang SS Nagasaka M. Spotlight on sotorasib (AMG 510) for KRAS G12C positive non-small cell lung cancer. Lung Cancer (Auckl) (2021) 12:115–22. doi: 10.2147/LCTT.S334623 PMC8504654 34675734

[27] Nakajima EC Drezner N Li X Mishra-Kalyani PS Liu Y Zhao H. FDA Approval summary: sotorasib for KRAS G12C-mutated metastatic NSCLC. Clin Cancer Res (2022) 28(8):1482–6. doi: 10.1158/1078-0432.CCR-21-3074 PMC9012672 34903582

[28] Roman M Baraibar I Lopez I Nadal E Rolfo C Vicent S. KRAS oncogene in non-small cell lung cancer: clinical perspectives on the treatment of an old target. Mol Cancer (2018) 17(1):33. doi: 10.1186/s12943-018-0789-x 29455666 PMC5817724

[29] Cinausero M Laprovitera N De Maglio G Gerratana L Riefolo M Macerelli M. KRAS and ERBB-family genetic alterations affect response to PD-1 inhibitors in metastatic nonsquamous NSCLC. Ther Adv Med Oncol (2019) 11:1758835919885540. doi: 10.1177/1758835919885540 31798692 PMC6859675

[30] Liu C Zheng S Jin R Wang X Wang F Zang R. The superior efficacy of anti-PD-1/PD-L1 immunotherapy in KRAS-mutant non-small cell lung cancer that correlates with an inflammatory phenotype and increased immunogenicity. Cancer Lett (2020) 470:95–105. doi: 10.1016/j.canlet.2019.10.027 31644929

[31] Salas-Benito D Pérez-Gracia JL Ponz-Sarvisé M Rodriguez-Ruiz ME Martínez-Forero I Castañón E. Paradigms on immunotherapy combinations with chemotherapy. Cancer Discovery (2021) 11(6):1353–67. doi: 10.1158/2159-8290.CD-20-1312 33712487

[32] Galluzzi L Humeau J Buqué A Zitvogel L Kroemer G. Immunostimulation with chemotherapy in the era of immune checkpoint inhibitors. Nat Rev Clin Oncol (2020) 17(12):725–41. doi: 10.1038/s41571-020-0413-z 32760014

[33] Galluzzi L Yamazaki T Kroemer G. Linking cellular stress responses to systemic homeostasis. Nat Rev Mol Cell Biol (2018) 19(11):731–45. doi: 10.1038/s41580-018-0068-0 30305710

[34] Galluzzi L Buqué A Kepp O Zitvogel L Kroemer G. Immunogenic cell death in cancer and infectious disease. Nat Rev Immunol (2017) 17(2):97–111. doi: 10.1038/nri.2016.107 27748397

[35] Uramoto H Onitsuka T Shimokawa H Hanagiri TTS. DHFR and GARFT expression in non-squamous cell carcinoma of NSCLC and malignant pleural mesothelioma patients treated with pemetrexed. Anticancer Res (2010) 30(10):4309–15. doi: 10.1097/CAD.0b013e32833db1bb 21036757

[36] Langer CJ Gadgeel SM Borghaei H Papadimitrakopoulou VA Patnaik A Powell SF. Carboplatin and pemetrexed with or without pembrolizumab for advanced, non-squamous non-small-cell lung cancer: a randomised, phase 2 cohort of the open-label KEYNOTE-021 study. Lancet Oncol (2016) 17(11):1497–508. doi: 10.1016/S1470-2045(16)30498-3 PMC6886237 27745820

[37] Ren Y Wang X Lou Z Huang S Zhuang H Wang Y. Induction of cell cycle arrest by increasing GTPRhoA levels *via* taxolinduced microtubule polymerization in renal cell carcinoma. Mol Med Rep (2017) 15(6):4273–9. doi: 10.3892/mmr.2017.6543 PMC5436224 28487984

[38] Khanna C Rosenberg M Vail DM. A review of paclitaxel and novel formulations including those suitable for use in dogs. J Vet Intern Med (2015) 29(4):1006–12. doi: 10.1111/jvim.12596 PMC4895360 26179168

[39] Adrianzen HD Ashai N Perez-Soler R Cheng H. Nanoparticle albumin bound-paclitaxel for treatment of advanced non-small cell lung cancer: an evaluation of the clinical evidence. Expert Opin Pharmacother (2019) 20(1):95–102. doi: 10.1080/14656566.2018.1546290 30439289

[40] Kundranda MN Niu J. Albumin-bound paclitaxel in solid tumors: clinical development and future directions. Drug Des Devel Ther (2015) 9:3767–77. doi: 10.2147/DDDT.S88023 PMC4521678 26244011

[41] Griesinger F Korol EE Kayaniyil S Varol N Ebner T Goring SM. Efficacy and safety of first-line carboplatin-versus cisplatin-based chemotherapy for non-small cell lung cancer: A meta-analysis. Lung Cancer (2019) 135:196–204. doi: 10.1016/j.lungcan.2019.07.010 31446995

[42] Zhou C Wu YL Chen G Liu X Zhu Y Lu S. BEYOND: A randomized, double-blind, placebo-controlled, multicenter, phase III study of first-line carboplatin/paclitaxel plus bevacizumab or placebo in Chinese patients with advanced or recurrent nonsquamous non-small-cell lung cancer. J Clin Oncol (2015) 33(19):2197–204. doi: 10.1200/JCO.2014.59.4424 26014294

[43] Elinav E Nowarski R Thaiss CA Hu B Jin C Flavell RA. Inflammation-induced cancer: crosstalk between tumours, immune cells and microorganisms. Nat Rev Cancer (2013) 13(11):759–71. doi: 10.1038/nrc3611 24154716

[44] Jiang T Qiao M Zhao C Li X Gao G Su C. Pretreatment neutrophil-to-lymphocyte ratio is associated with outcome of advanced-stage cancer patients treated with immunotherapy: a meta-analysis. Cancer Immunol Immunother (2018) 67(5):713–27. doi: 10.1007/s00262-018-2126-z PMC11028313 29423649

[45] Parikh K Kumar A Ahmed J Anwar A Puccio C Chun H. Peripheral monocytes and neutrophils predict response to immune checkpoint inhibitors in patients with metastatic non-small cell lung cancer. Cancer Immunol Immunother (2018) 67(9):1365–70. doi: 10.1007/s00262-018-2192-2 PMC11028276 29968154

[46] Tanizaki J Haratani K Hayashi H Chiba Y Nakamura Y Yonesaka K. Peripheral blood biomarkers associated with clinical outcome in non-small cell lung cancer patients treated with nivolumab. J Thorac Oncol (2018) 13(1):97–105. doi: 10.1016/j.jtho.2017.10.030 29170120

[47] Kargl J Busch SE Yang GH Kim KH Hanke ML Metz HE. Neutrophils dominate the immune cell composition in non-small cell lung cancer. Nat Commun (2017) 8:14381. doi: 10.1038/ncomms14381 28146145 PMC5296654

[48] Zuazo M Arasanz H Bocanegra A Fernandez G Chocarro L Vera R. Systemic CD4 immunity as a key contributor to PD-L1/PD-1 blockade immunotherapy efficacy. Front Immunol (2020) 11:586907. doi: 10.3389/fimmu.2020.586907 33329566 PMC7734243

[49] Farhood B Najafi M Mortezaee K. CD8+ cytotoxic T lymphocytes in cancer immunotherapy: A review. J Cell Physiol (2019) 234(6):8509–21. doi: 10.1002/jcp.27782 30520029

[50] Souto JC Vila L Bru A. Polymorphonuclear neutrophils and cancer: intense and sustained neutrophilia as a treatment against solid tumors. Med Res Rev (2011) 31(3):311–63. doi: 10.1002/med.20185 19967776

